# Supplementary material for: Co-Designing Digital Technologies for Improving Clinical Care in People with Parkinson’s Disease: What Did We Learn?
Source: Sensors (Basel). 2023 May 22;23(10):4957. doi: 10.3390/s23104957 (PMC10222343; doi:10.3390/s23104957)
Supplement: Supplementary file 1 [file sensors-23-04957-s001.zip › sensors-2308859-supplementary.pdf]

Supplementary Table S1. Illustrative quotes:

**Theme/category**	**Illustrative quote**
General utilisability and Usability	“Very useful and accurate. Especially convenient for Parkinson’s disease patients living on small cities and villages” (Patient 13)<br>“Perhaps for older people unfamiliar with the technology a bit more complicated”. (Patient 19)<br>“The use of this tool can be effective as a guiding or complementary tool supervised by a professional.” (Patient 24)<br>“If this system was used prior to my physician's appointment it would be more helpful. (Patient 17)
Remote use	“It helps me to see my status easily. I can do it myself” (Patient 20)<br>“Will have to set time aside, maybe once a week. Not so much time can be spent using the App if you are still working” (Patient 17)<br>“If it is improved. It would be a better recorder in portions of remote visit with the doctor.” (Patient 22)”
Positive comments	“I liked the fact that i had autonomy and didn't need anyone to monitor me” (Patient 16)<br>“Improved study of the disease” (patient 3)<br>“Explicit explanations (Patient 15)”
Negative comments	“I think to encourage use it should be more fun (patient 6)<br>“The instructions are not aligned with the text. What he (N.B: the person speaking) is saying is not the same and the sound is not loud. (Patient 12)”<br>“It is not a great thing for self-management but may be for doctor's management (Patient 22)”

Supplementary Figure S1. *MooVeo* Report

Participant ID: 1903
Test date: 26/09/2020
Test time: 20:51

MooVeo Report

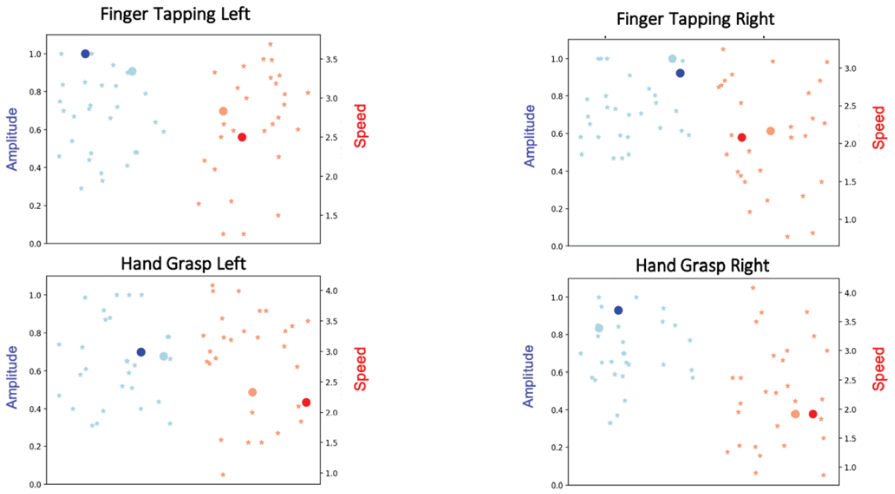

	Current Score	Norm
Speed (taps/sec)	2.33	2.76(2.55-2.97)
Speed Variation	0.07	0.28
Amplitude (°/sec)	0.85	0.65(0.6-0.71)
Amplitude Variation	0.18	0.32
Fatigue	0.25	0.06(-0.0-0.12)
Fatigue Variation	0.27	3.87

	Current Score	Norm
Speed (taps/sec)	2.0	2.19(1.97-2.4)
Speed Variation	0.04	0.36
Amplitude (°/sec)	0.93	0.71(0.66-0.75)
Amplitude Variation	0.0	0.24
Fatigue	0.1	0.02(-0.03-0.06)
Fatigue Variation	0.27	9.38

Change from previous evaluation

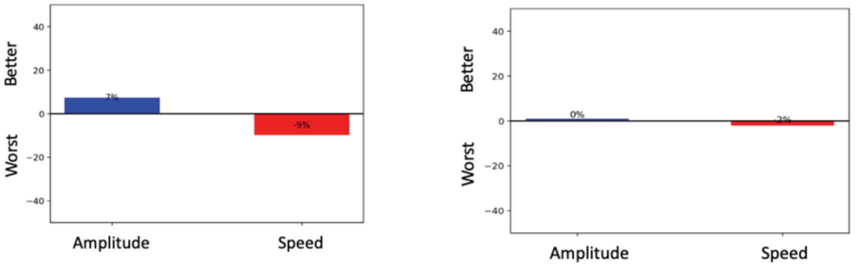

MooVeo Score: X.XX (Probability of Parkinson's Disease)

Page 1

Supplementary Figure S1. Illustrative example of the patient's report generated by *MooVeo*. It includes a graphic representation of the amplitude and speed of movement of the finger tapping and hand grasp bradykinesia tasks, the raw values of some movement characteristics extracted by the system (ref), and a comparison with the previous evaluation. The *MooVeo* Platform immediately generates the report and is accessible for downloading by the user.
